# Semi-PBPK Modeling and Simulation to Evaluate the Local and Systemic Pharmacokinetics of OC-01(Varenicline) Nasal Spray

**DOI:** 10.3389/fphar.2022.910629

**Published:** 2022-07-07

**Authors:** Xiaofei Wu, Fan Zhang, Mengyang Yu, Faming Ding, Jinghui Luo, Bo Liu, Yuan Li, Zhiping Li, Hongyun Wang

**Affiliations:** ^1^ Clinical Pharmacology Research Center, State Key Laboratory of Complex Severe and Rare Diseases, NMPA Key Laboratory for Clinical Research and Evaluation of Drug, Beijing Key Laboratory of Clinical PK & PD Investigation for Innovative Drugs, Peking Union Medical College Hospital, Chinese Academy of Medical Sciences and Peking Union Medical College, Beijing, China; ^2^ Ji Xing Pharmaceuticals (Shanghai) Co., Ltd., Shanghai, China

**Keywords:** nasal spray, varenicline, OC-01, dry eye disease, pharmacokinetics, semi-PBPK model

## Abstract

This study aimed to build a nasal semi-physiologically based pharmacokinetic (PBPK) model to predict the intranasal pharmacokinetic (PK) of the OC-01(varenicline) nasal spray and accelerate the development of this drug. Based on the physiology of the human upper respiratory system, the semi-PBPK model was established and validated using systemic plasma PK data of varenicline previously observed in Americans and Chinese. Drug concentrations, both in respiratory tissue and plasma circulation system, were well simulated, and it was indicated that local concentration at the target site (nasal cavity) was significantly higher than that of plasma when OC-01 nasal spray was administered. The nasal semi-PBPK model successfully depicted the absorption and distribution of intranasal varenicline in the respiratory tissues and provided an alternative to clinical PK study of OC-01 nasal spray in Chinese. Meanwhile the current study presented a viable framework for predicting respiratory concentrations for other novel nasal spray drugs by semi-PBPK modeling.

## 1 Introduction

OC-01 (varenicline) nasal spray was developed by Oyster Point Pharmaceuticals for the treatment of signs and symptoms of dry eye disease (DED). The active substance of OC-01 nasal spray is varenicline tartrate, a small molecule nicotinic acetylcholine receptor (nAChR) agonist, and its oral formulation (Chantix^®^/Champix^®^) is currently marketed as an aid to smoking cessation.

The nAChRs are a class of pentameric ligand-gated ion channels with high affinity and selectivity for both nicotine and acetylcholine, and nAChRs comprise combinations of α- and β-subunits. Varenicline is a partial agonist of the human α_4_β_2_ and α_4_α_6_β_2_ nAChRs, a potent agonist of the α_7_ nAChR, and a moderate agonist of the α_3_β_4_ and α_3_α_5_β_4_ nAChR subtype. It is hypothesized that the primary nAChR subtypes instigating the trigeminal parasympathetic pathway (TPP) are the α_4_β_2_ and α_4_α_6_β_2_ nAChRs located on the trigeminal nerve endings in the nasal mucosa. When delivered as a nasal spray, varenicline activates the TPP and stimulates lacrimal functional units (LFU) to promote natural tear film production, improving the signs and symptoms of DED (Nau et al., 2021; Quiroz-Mercado et al., 2021; Wirta et al., 2021). To date, several clinical studies of OC-01 nasal spray have been conducted to evaluate the safety, efficacy, and pharmacokinetic (PK) of OC-01 in DED treatment (ClinicalTrials.gov, 2021a; ClinicalTrials.gov, 2021b; ClinicalTrials.gov, 2021c; ClinicalTrials.gov, 2021d; ClinicalTrials.gov, 2021e; ClinicalTrials.gov, 2021f). In October 2021, the US Food and Drug Administration (FDA) approved OC-01 (varenicline) nasal spray (0.03 mg/spray/nostril, at a total dose of 0.06 mg per delivery, BID, hereafter referred to as 0.06 mg BID) for the treatment of signs and symptoms of DED.

For the locally acting drugs with known active ingredients, such as nasal sprays, understanding the PK characteristics of local sites is crucial for exploring the safety and efficacy of drugs, as well as the decision on subsequent clinical trials. Nevertheless, due to technical or ethical issues, it is tough and sometimes impractical to measure the concentration of human local organs in clinical studies. As for OC-01 nasal spray, the pharmacokinetics in Americans was evaluated using plasma concentrations but not local intranasal tissue concentrations (Nau et al., 2021). It was found that intranasal varenicline, when administered at its highest intended dose of 0.12 mg in nasal spray, delivered only 7% to the systemic circulation compared with oral varenicline at the dose of 1 mg. Currently OC-01 nasal spray is applied for approval in China. Of note varenicline tablet has been approved by National Medical Products Administration (NMPA) as a smoking cessation treatment in 2008 (Faessel et al., 2010), and the safety of the active ingredient (varenicline) has been proved (Xiao et al., 2009; Nau et al., 2021). Two questions remain: 1) Is it necessary to assess the system PK characteristic following the intranasal administration of OC-01 nasal spray in Chinese population? 2) Are there any alternative and/or feasible technologies to evaluate the target tissue concentration? In fact, there are common limitations in the clinical evaluation of locally administered drugs, including the inability to determine the drug exposure at the site where drugs will be acting to produce either therapeutic or toxic effects, and in some cases, systemic concentrations at clinical doses are not measurable by routine analytical procedures (US Food and Drug Administration, 2002). These problems make the development of local acting drugs very challenging and time-consuming. For these reasons, new methods were developed to make the development more cost-and time-effective, such as clinically relevant *in vitro* tools, physiology-based pharmacokinetic (PBPK) modeling and so on (US Food and Drug Administration, 2016).

PBPK modeling is a key component of model-based drug development and is increasingly embraced by industry and regulatory authorities (Wang et al., 2016). It integrates diverse information on mechanism of action, anatomy, physiology, biochemistry, and metabolism and enables the prediction of system and tissue-specific exposure to the drug against time to depict the PK profiles (Polak et al., 2019). The simulation can illustrate the PK characteristics among different populations (Cui et al., 2020) and avoid unnecessary clinical trials. Several PBPK models have been established to predict the local tissue pharmacokinetics (Plowchalk et al., 1997; Andersen et al., 2002; Sarangapani et al., 2002; Gaohua et al., 2015; Le Merdy et al., 2020). In the study reported by Sarangapani et al. (2002), a PBPK model was developed to predict drug concentration in blood, liver, and respiratory tract tissue in rats. Gaohua et al. (2015) developed a multicompartment lung model and predicted pulmonary pharmacokinetics of the antituberculosis drug in cells. In this study, for the first time, a nasal spray semi-PBPK model was established and used to depict the absorption and distribution of intranasal varenicline in different tissues of the respiratory system so as to guide the clinical trial of OC-01 nasal spray in Chinese. We hope this model can support the phase I–III clinical studies of OC-01 nasal spray in Chinese and also provide a framework for predicting respiratory concentrations of novel nasal spray drugs.

## 2 Methods

### 2.1 Data Source

The plasma PK data of intranasal varenicline 0.12 mg (nasal spray) and oral varenicline 1 mg (tablet) in American subjects came from the phase I clinical study (Nau et al., 2021). The plasma PK data of a single oral administration of 1 mg varenicline (tablets) in Chinese subjects came from the study of Y. Xiao et al. (2009). The model was fitted to plasma concentrations at various time points.

### 2.2 Software

The semi-PBPK model was established using B_2_O simulator software (version 2.0, Hubei Yinghan Pharmaceutical Technology Co., Ltd, China), a virtual drug development platform that integrates formulation development, drug–drug interaction (DDI), and other characteristic function modules. All image data were collected using the Digitizer tool in Origin (version 2018, Origin Lab, United States), and the noncompartmental analysis (NCA) was performed using Phoenix WinNonlin 8.1 (Certara LP, Princeton, NJ, United States).

### 2.3 Model Structure

**FIGURE 1:**
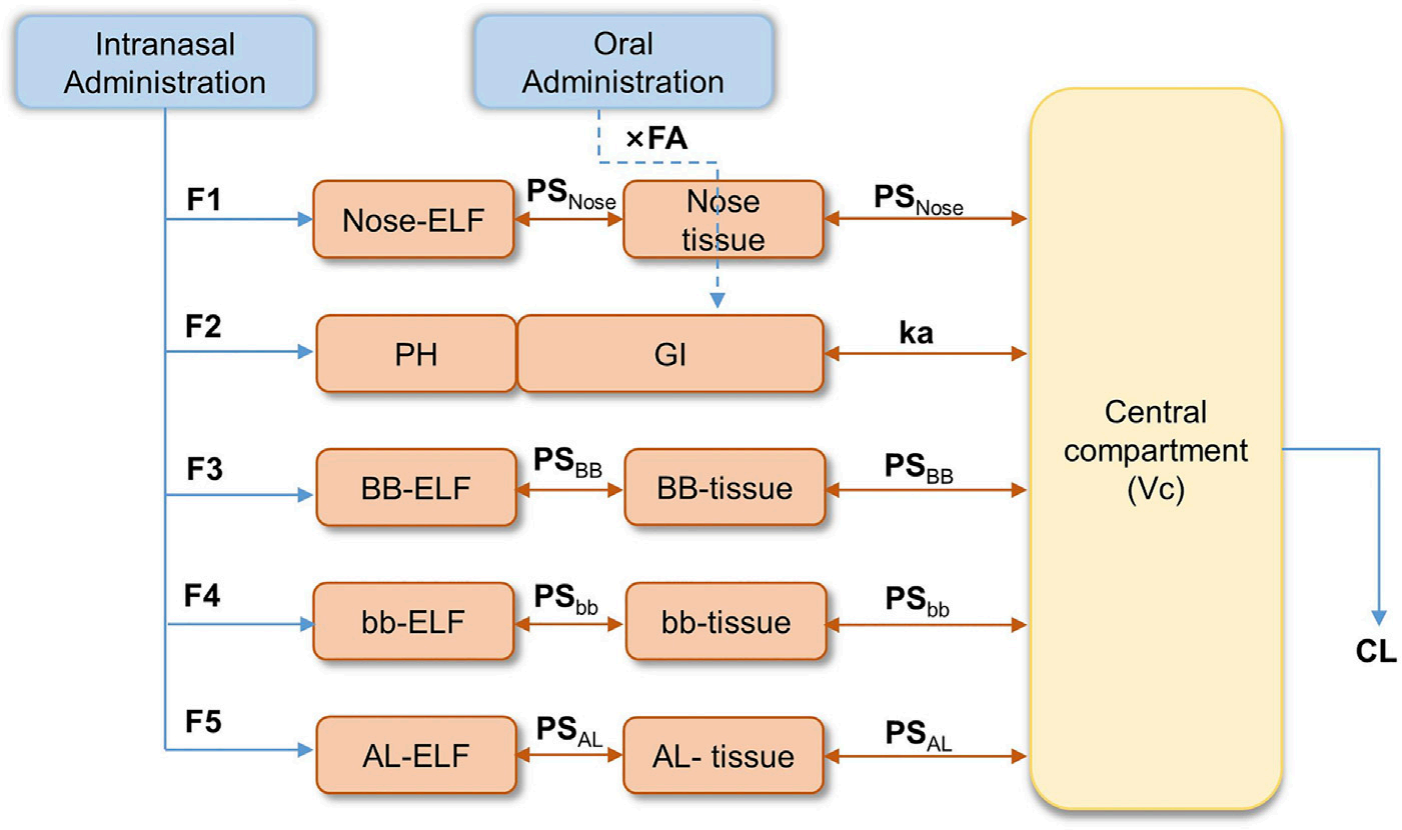
Semi-PBPK model structure for the human respiratory system. (F1-F5: fraction of drug entering the epithelial lining fluid (ELF); Nose-ELF: nasal cavity; PH: pharynx; BB-ELF: bronchi; bb-ELF: bronchioles; AL-ELF: alveoli; PS: permeation elimination rate; PS_Nose_: PS in the nasal cavity; PS_BB_: PS in bronchi; PS_bb_: PS in bronchioles; PS_AL_: PS in alveoli; K_mcc, bb_: the peristalsis of respiratory epithelial cilia between bb-ELF and BB-ELF; K_mcc, BB_: the peristalsis of respiratory epithelial cilia between BB-ELF and PH.).

The semi-PBPK model was constructed based on the physiology of the human respiratory system. Organs other than the respiratory system were simplified as a single compartment. The structure of the mechanistic multicompartment nose model is shown in Figure 1.

Based on the effect of particle sizes in the spray and the fractions of varenicline entering the epithelial lining fluid (ELF), the system was described by five segments representing nasal cavity (Nose-ELF), pharynx (PH), bronchi (BB-ELF), bronchioles (bb-ELF), and alveoli (AL-ELF), respectively. The fraction of drugs entering the ELF was described as F1–F5 in Figure 1. Orally administered varenicline were considered directly entering the gastrointestinal (GI) tract (Figure 1). The model assumed that varenicline in each segment of ELF, except PH, entered the corresponding tissue segments through permeation and then permeated into the central systemic circulation. The permeation elimination rate (PS) was described as PS_Nose_, PS_BB_, PS_bb,_ and PS_AL_, representing the PS in the nasal cavity, bronchi, bronchioles, and alveoli, respectively. K_mcc_,_bb_ and K_mcc,BB_ were the clearance of the ELF associated with peristalsis of respiratory epithelial cilia between bb-ELF and BB-ELF and between BB-ELF and PH. Varenicline entering the pharynx was swallowed into the gastrointestinal tract directly.

The absorption process of the drug entering GI was described using the first-order absorption model. In Figure 1, ka was the gastrointestinal absorption rate of the orally administered drug, and FA was the absorbed fraction of the drug after entering GI. Since the model did not consider the first-pass effect, FA was approximate to bioavailability. Based on previous results, FA was tentatively set to 0.9 (Faessel et al., 2010). Additionally, considering the bioequivalence of oral PK between Americans and Chinese, FA in the Chinese population was also assumed to be 0.9. After absorption by the respiratory tract or the gastrointestinal tract, varenicline entered the systemic circulation, where Vc was the volume and CL was the varenicline clearance. The equations used to describe the drug concentrations within the model were described in full in Supplementary Tables S1–S3.

### 2.4 Simulation Strategies

**FIGURE 2:**
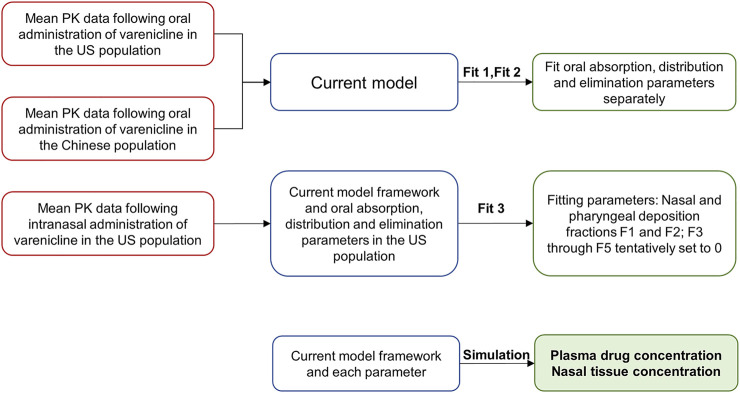
Simulation strategies.

Three simulation steps based on the semi-PBPK model were performed to simulate the nasal PK characteristics following intranasal administration in American and Chinese subjects. The steps included simulating oral administration data in Americans (Fit 1), simulating oral administration data in Chinese (Fit 2), and simulating intranasal administration data in Americans (Fit 3). The overall framework is shown in Figure 2.

Parameters like gastrointestinal absorption, distribution, and elimination were determined in Fit 1 and Fit 2 (Figure 2) by fitting oral PK data obtained from Americans and Chinese to the semi-PBPK model.

Fit 3 was used to determine deposition fractions of varenicline in different segments following intranasal administration, using the plasma PK data following intranasal administration in Americans. In the study reported by Cheng (Cheng, 2014), the deposition of nasal spray with a particle size of 0.001–10 μm in different segments of the respiratory tract following intranasal administration was studied. It can be deduced that when the particle size is 10 μm or larger, very little will be deposited in such segments as BB, bb, and AL. Considering that the particles’ median sizes in the nasal spray are typically around 30–120 μm (Salar-Behzadi et al., 2017), F3, F4, and F5 were preliminarily set to 0 in the model.

After fitting each parameter, local and systemic PK of varenicline following intranasal administration can be obtained by fitting parameters to the semi-PBPK model to study the difference between the two populations.

### 2.5 Modeling and Simulation Process

#### 2.5.1 Determination of Fixed Parameters

Model parameters (Andersen et al., 2002; Faessel et al., 2010; Gaohua et al., 2015) are listed in Table 1. They were assumed to be the same across different populations. Other physiological parameters of the respiratory tract were calculated based on physiological data, including volumes of ELF and tissue of a respiratory tract segment x (V_x-ELF_ and V_x-tissue_), the permeation elimination rate (PS_x_) between ELF and tissue, and that between tissue and the central compartment. Here, x was nose, BB, bb, or AL. The systemic availability following gastrointestinal absorption of oral varenicline could reach about 90% (Faessel et al., 2010). Therefore, the FA in the semi-PBPK model in this report was set to a fixed value of 0.9.

**TABLE 1:** The fixed parameters of the semi-PBPK model.

Parameter	Value	Unit	Definition	Source
FA	0.900		Fraction of absorbed drug following entry into the gastrointestinal tract	Reference, Xiao et al. (2009)
f_u, ELF_	1		Fraction of unabsorbed drug in the epithelial lining fluid	Default
f_u,tissue_	1		Fraction of unabsorbed drug in the respiratory tract tissue	Default
f_u,p_	0.800		Fraction of unabsorbed drug in plasma	Reference, Faessel et al. (2010)
k_mcc,bb_	0.083	h^−1^	Elimination rate due to bronchiolar ciliary peristalsis	Reference, Plowchalk et al. (1997)
k_mcc,BB_	0.417	h^−1^	Elimination rate due to bronchial ciliary peristalsis	Reference, Plowchalk et al. (1997)
V_Nose-ELF_	2.459*10^–4^	L	The volume of nasal epithelial lining fluid	Reference, Polak et al. (2019)
V_BB-ELF_	0.0021	L	The volume of bronchial epithelial lining fluid	Reference, Plowchalk et al. (1997)
V_bb-ELF_	0.0021	L	The volume of bronchiolar epithelial lining fluid	Reference, Plowchalk et al. (1997)
V_AL-ELF_	0.0208	L	The volume of alveolar epithelial lining fluid	Reference, Plowchalk et al. (1997)
V_Nose-tissue_	2.828*10^–3^	L	The volume of nasal mucosal tissue	Reference, Polak et al. (2019)
V_BB-tissue_	0.038	L	Volume of bronchial mucosal tissue	Reference, Plowchalk et al. (1997)
V_bb-tissue_	0.038	L	Volume of bronchiolar tissue	Reference, Plowchalk et al. (1997)
V_AL-tissue_	0.381	L	The volume of alveolar tissue	Reference, Plowchalk et al. (1997)
PS_Nose_	0.0045	L/h	The permeation elimination rate of the nasal mucosa	Calculated
PS_BB_	0.138	L/h	Permeation elimination rate of bronchial mucosa	Calculated
PS_bb_	0.138	L/h	Permeation elimination rate of bronchiolar mucosa	Calculated
PS_AL_	25.728	L/h	The permeation elimination rate of the alveolar inner wall	Calculated

Because plasma protein binding rate of varenicline is lower than 20% (Faessel et al., 2010), f_u,p_ was set to a fixed value of 0.8. Additionally, given the lack of proteins that can specifically bind to the drug in ELF and the inadequacy of information regarding the protein binding rate of varenicline in respiratory tract tissues, both f_u, ELF_ and f_u, tissue_ of varenicline were assumed to be 1. f_u, ELF_, f_u, tissue_, and f_u, p_ were the free fraction of drug in ELF, tissue, and plasma, respectively. In Table 1, ELF volumes of different segments of the respiratory tract (excluding pharynx PH) are V_Nose-ELF_, V_BB-ELF_, V_bb-ELF_, and V_AL-ELF_. Tissue volumes of different segments were V_Nose-tissue_, V_BB-tissue_, V_bb-tissue_, and V_AL-tissue_. Permeation elimination rates between ELF and tissue of each segment and between tissue and the central compartment were PS_Nose_, PS_BB_, PS_bb_, and PS_AL_. And the parameters of V_Nose-ELF_ and V_Nose-tissue_ were calculated based on the internal surface area of the nasal cavity (S_Nose_), nasal mucosal thickness (H_Nose_), and nasal tissue thickness (D_Nose_):

$$V_{Nose-ELF}=S_{Nose}*H_{Nose}$$ (1)

$${\text{V}}_{\mathrm{Nose}-\mathrm{tissue}}={\text{S}}_{\mathrm{Nose}}*{\text{D}}_{\mathrm{Nose}}$$ (2)

Here, S_Nose_ was 0.0246 m^2^, H_Nose_ was 10^–6^ m, and D_Nose_ was 1.15*10^–4^ m (Andersen et al., 2002). Other parameters like volumes of ELF and tissue of each segment of the respiratory tract were derived from the study conducted by Gaohua et al. (2015). The surface area of each segment (surface areas of BB and bb S_BB_ = S_bb_ = 0.75 m^2^, the AL segment S_AL_ = 140 m^2^) was also used to calculate each segment’s PS.

Similarly, the permeation elimination rate between ELF and tissue of respiratory tract mucosa and that between tissue and the central compartment could also be calculated based on the surface area and the effective permeation coefficient (P_eff_). Since the P_eff_ value of varenicline in the respiratory tract was not available, it was estimated based on data of other drugs. SalarBehzadi et al. calculated the P_eff_ value of budesonide in the upper respiratory tract using multiple approaches (Salar-Behzadi et al., 2017), and the mean value was 2.228*10^–6^ cm/s. This study calculated the P_eff_ value of varenicline based on budesonide’s respiratory tract P_eff_ value and the *in vitro* apparent permeation coefficients (P_app_) of the two drugs. Ideally, the P_app_ to the respiratory tract P_eff_ should be based on permeation test data in the Calu-3 cell line. However, as only data in the Caco-2 cell line were available for varenicline, the respiratory tract P_eff_ of varenicline was estimated based on the mean P_app_ values of varenicline and budesonide in the Caco-2 cell line [26.145*10^–6^ cm/s (US Food and Drug Administration, 2005) and 11.4*10^–6^ cm/s (Dilger et al., 2004)]:

$$P_{eff,\;varenicline\;}=\frac{P_{app,\;varenicline}}{P_{app,\;budesonide}}*P_{eff,\;budesonide}=\frac{\mathrm{26}\mathrm{.145}*10^{-6}}{\mathrm{11}\mathrm{.4}*10^{-6}}*2\mathrm{.228}*10^{-6}\approx 5\mathrm{.110}*10^{-6}\text{cm/s}$$ (3)

In this study, the PS in each segment was calculated using the equation below. Here the parameter of S was the surface area of each segment of the respiratory (see Table 1 for the results):

$$PS=P_{eff,\;varenicline\;}*S$$ (4)

#### 2.5.2 Fit 1 and Fit 2

The model fit the average plasma PK data of varenicline following a single-dose oral administration in Americans (Fit 1) and Chinese (Fit 2). Parameters like the delay time of gastrointestinal absorption (t_lag_), ka, Vc, and CL in Americans and Chinese were obtained.

#### 2.5.3 Fit 3

Based on t_lag_, ka, Vc, and CL in Americans obtained in Fit 1, PK data of intranasal administration in ZEN study (Nau et al., 2021) were fitted, fractions entered the nasal cavity, and the pharynx (F1–F2) were obtained. Additionally, the fractions were assumed to be equal between Americans and Chinese.

#### 2.5.4 Simulation Evaluation

In the above model framework, the semi-PBPK models of intranasal administration for Americans and Chinese were separately established based on t_lag_, ka, Vc, and CL in the two populations and using the same F1 and F2. The coefficient of variation was introduced to the distribution and elimination compartment. The coefficients of CL and V variation were set to the default value of 38%. Varenicline concentrations in the central compartment following intranasal and oral administration in 300 Chinese or American virtual subjects were simulated in the model. The corresponding individual or mean PK data measured were compared to check whether the model matched with measured values.

### 2.6 Simulation of Multidose Administration and Statistical Analysis of Pharmacokinetics

PK results following single and multidose intranasal administration in Chinese and American virtual subjects (*n* = 500) were simulated using the model in this study. Systemic and nasal drug concentration–time curves and time to steady-state in two populations were plotted. The administration frequency and dosage followed the FDA label (US Food and Drug Administration, 2005). The simulation of single-dose administration included PK data 72 h following single-dose administration. Regarding multidose administration, PK following administration for seven consecutive days was simulated. Only one dose was administered on day 7, and PK 72 h later was simulated.

After simulation, systemic and nasal PK parameters in American and Chinese subjects were calculated and compared to assess the potential PK difference between the two populations. The following parameters were calculated using the NCA method in Phoenix WinNonlin 8.1 (Certara LP, Princeton, NJ, United States): AUC_0-12_, area under the plasma concentration–time curve from the start of the last dose to 12 h; AUC_0-∞_, area under the plasma concentration–time curve from the start of the last dose to infinity; C_max_, maximum measured plasma concentration; T_max_, time to the maximum measured plasma concentration; CL/F, Oral clearance; V/F, apparent volume of distribution; T_1/2_, elimination half-life; R_Cmax_, C_max_ at day 7/C_max_ at day1; and R_AUC_, AUC_0-12_ at day 7/AUC_0-12_ at day 1.

## 3 Results

### 3.1 Fit 1 and Fit 2

**FIGURE 3:**
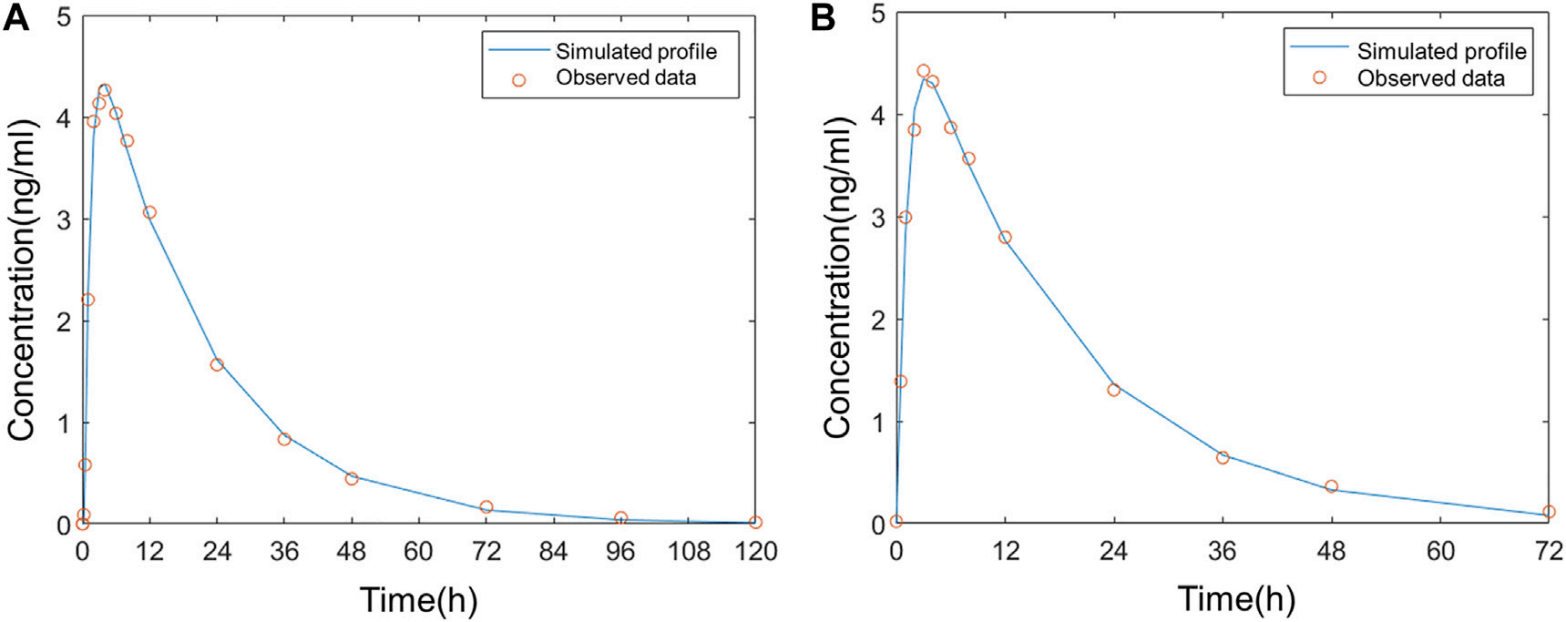
Comparison of the observed plasma concentration–time curve following oral administration to the simulated **(A)** in Americans; **(B)** in Chinese.

The model was fitted to the average PK parameters of varenicline following single-dose oral administration in Americans (Fit 1) and Chinese (Fit 2), and the results are shown in Figure 3. The parameter estimates are summarized in Table 2 and were used for the following modeling. k_12_, k_21_, k_13_, and k_31_ were all set to a fixed value of 0.

**TABLE 2:** PK parameter values obtained by model fitting in Fit 1 and Fit 2.

Parameter	Result of fit 1	Result of fit 2	Unit
t_lag_	0.371	0.156	h
ka	1.036	1.064	h^−1^
Vc	194.352	190.823	L
CL	10.001	11.320	L/h

t_lag_, the delay time of gastrointestinal absorption; ka, absorption rate constant; Vc, distribution volume of central compartment; CL, clearance.

### 3.2 Fit 3

**FIGURE 4:**
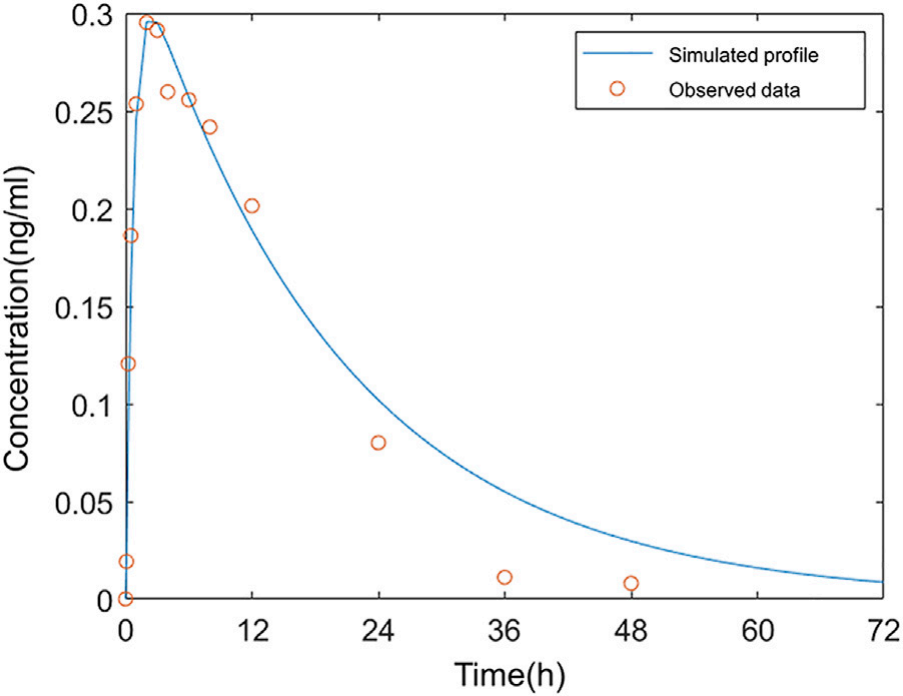
Comparison of the observed concentration–time curve following intranasal administration in Americans to the simulated.

The average plasma PK parameters following intranasal administration of 0.12 mg varenicline in Americans were fitted to the model, and the results are shown in Figure 4. Based on the droplet particle size and distribution fractions calculated by Cheng (Cheng, 2014), the deposition fractions F3 in BB, F4 in bb, and F5 in AL of the lower respiratory tract were all set to a fixed value of 0. The value of F1 in the nose is 0.547, and F2 in the pharynx is 7.6375 *10^–7^.

The simulation results showed that 54.7% of the administered dose was distributed on the inner wall of the nasal cavity. The fraction deposited at the pharynx was approximate 0. The very low F2 indicated that varenicline hardly entered the gastrointestinal tract following intranasal administration. The drug droplets were primarily retained in the nasal passage. In the study reported by Frank et al., 100% of droplets larger than 10 μm were deposited in the nasal passage (Frank et al., 2012). The sum of F1 and F2 did not reach 100% because the nasal compartment of the model in this study only covered mucosal parts of the nasal cavity that can absorb drugs. The fraction deposited in the nasal vestibule (covered with keratinized skin) was not included. A deposition fraction of 54.7% in the nasal cavity, excluding the nasal vestibule, matches with the simulation study of the nasal spray deposition in the respiratory system reported by Keeler et al. (2016).

The simulation results matched with the measured plasma concentration data except three values after 24 h were below the fitted curve. According to the clinical study of ZEN (Nau et al., 2021), all values below the lower limit of quantitation (LLOQ) were regarded as 0 in calculating arithmetic means. As a result, the mean measured concentration values were below the simulated plasma concentrations in this period. The deviations from measured values at these data points on fitted curves in Fit 3 were acceptable.

### 3.3 Simulation Evaluation

**FIGURE 5:**
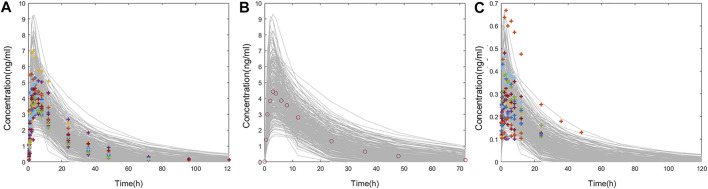
**(A)** Comparison of individual PK data following oral administration of 1 mg varenicline in Americans (colored cross) and the concentration–time curve in 300 American virtual subjects (gray line). **(B)** Comparison of mean PK data following oral administration of 1 mg varenicline in Chinese (red circle) and the concentration–time curve in 300 Chinese virtual subjects (gray line). **(C)** Comparison of individual PK data following intranasal administration of 0.12 mg varenicline in Americans (colored cross) and the concentration–time curve in 300 American virtual subjects (gray line).

In this study, fixed parameters (Table 1) and results of Fit 3 were shared for Americans and Chinese, and the main difference was focused on t_lag_, ka, Vc, and CL in Fit 1 and Fit 2 (Table 2). Comparison between simulated data and the actual average measured values (Chinese population) or individual measured values (American population) are presented in Figure 5. The corresponding PK parameters of simulated and observed values included C_max_, AUC_0-t_, AUC_0-∞_, and T_1/2_, the median, minimum, and maximum of time to the maximum measured plasma concentration are listed and compared in Table 3, Table 4, and Table 5.

**TABLE 3:** Comparison of measured PK values following oral administration of 1 mg varenicline in Americans and simulated PK values in 300 American virtual subjects.

Parameter	Simulated value			Parameter	Measured value		
	Mean	Standard error	Coefficient of variation (%)		Mean	Standard error	Coefficient of variation (%)
C_max_ (ng/ml)	4.58	1.48	32.40	C_max_ (ng/ml)	4.63	2.02	53.00
AUC_0-t_ (h*ng/mL)	103.73	37.54	36.19	AUC_0-t_ (h*ng/mL)	98.74	25.49	25.80
AUC_0-∞_ (h*ng/mL)	105.80	40.35	38.14	AUC_0-∞_ (h*ng/mL)	102.53	26.82	26.20
T_1/2_ (h)	15.05	8.15	54.13	T_1/2_ (h)	19.59	10.39	53.00
	**Median**	**Minimum**	**Maximum**		**Median**	**Minimum**	**Maximum**
T_max_ (h)	4.00	2.00	6.00	T_max_ (h)	3.00	1.00	6.00

C_max_, maximum concentration; AUC_0-t,_ area under the plasma concentration *vs*. time curve from the start of the last dose to the last time at which there is a quantifiable concentration; AUC_0-∞_, AUC_0-t_ extrapolated to infinity; T_1/2_, apparent terminal elimination half-life; T_max_, time to C_max_.

**TABLE 4:** Comparison of measured PK values following oral administration of 1 mg varenicline in Chinese and simulated PK values in 300 Chinese virtual subjects.

Parameter	Simulated value			Parameter	Measured value		
	Mean	Standard error	Coefficient of variation (%)		Mean	Standard error	Coefficient of variation (%)
C_max_ (ng/ml)	4.59	1.47	32.06	C_max_ (ng/ml)	4.95	0.69	14.00
AUC_0-t_ (h*ng/mL)	88.73	30.14	33.97	AUC_0-t_ (h*ng/ml)	87.31	19.85	23.00
AUC_0-∞_ (h*ng/mL)	93.55	35.68	38.14	AUC_0-∞_ (h*ng/ml)	91.36	21.23	23.00
T_1/2_ (h)	13.07	7.07	54.14	T_1/2_ (h)	15.23	3.38	22.00
	**Median**	**Minimum**	**Maximum**		**Median**	**Minimum**	**Maximum**
T_max_ (h)	3.00	2.00	4.00	T_max_ (h)	3.00	0.50	6.00

C_max_, maximum concentration; AUC_0-t,_ area under the plasma concentration *vs*. time curve from the start of the last dose to the last time at which there is a quantifiable concentration; AUC_0-∞_, AUC_0-t_ extrapolated to infinity; T_1/2_, apparent terminal elimination half-life, T_max_, time to C_max_.

**TABLE 5:** Comparison of measured PK values following intranasal administration of 0.12 mg varenicline in Americans and simulated PK values in 300 American virtual subjects.

Parameter	Simulated value			Parameter	Measured value		
	Mean	Standard error	Coefficient of variation (%)		Mean	Standard error	Coefficient of variation (%)
C_max_ (ng/ml)	0.32	0.11	33.68	C_max_ (ng/ml)	0.34	0.13	37.60
AUC_0-t_ (h*ng/mL)	6.81	2.47	36.20	AUC_0-t_ (h*ng/ml)	4.49	3.42	76.20
AUC_0-∞_ (h*ng/mL)	6.95	2.65	38.11	AUC_0-∞_ (h*ng/ml)	8.30	4.09	49.30
T_1/2_ (h)	15.05	8.14	54.09	T_1/2_ (h)	18.93	9.90	52.30
	**Median**	**Minimum**	**Maximum**		**Median**	**Minimum**	**Maximum**
T_max_ (h)	2.00	2.00	3.00	T_max_ (h)	2.00	0.30	3.00

C_max_, maximum concentration; AUC_0-t,_ area under the plasma concentration *vs*. time curve from the start of the last dose to the last time at which there is a quantifiable concentration; AUC_0-∞_, AUC_0-t_ extrapolated to infinity; T_1/2_, apparent terminal elimination half-life, T_max_, time to C_max_.

According to the measured and simulated concentration–time curves and PK parameters, it was clear that the simulated values obtained with the model matched well with the measured values. Among the PK parameters, the simulated values of T_1/2_ were slightly lower than measured values; the differences were associated with the coefficient of variation of the model. Considering the significant coefficient of variation of measured T_1/2_ values in the previous studies (Xiao et al., 2009; Nau et al., 2021), simulated values obtained with the model reasonably matched with measured values.

### 3.4 Simulation of Multidose Administration and Statistical Analysis of Pharmacokinetics

**FIGURE 6:**
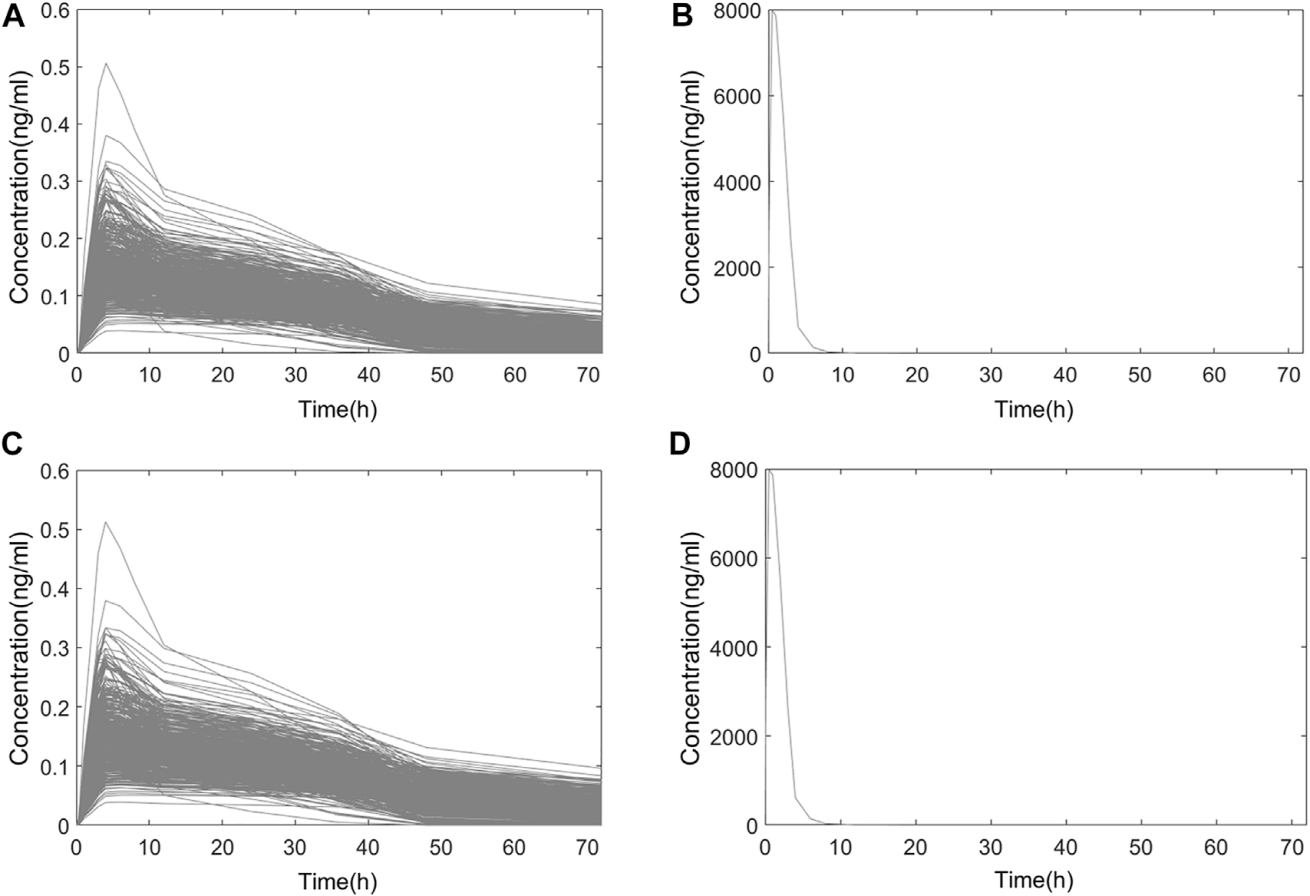
Simulated PK curve of single-dose administration. **(A)** Systemic PK in Chinese; **(B)** nasal PK in Chinese; **(C)** systemic PK in Americans; **(D)** nasal PK in Americans.

**FIGURE 7:**
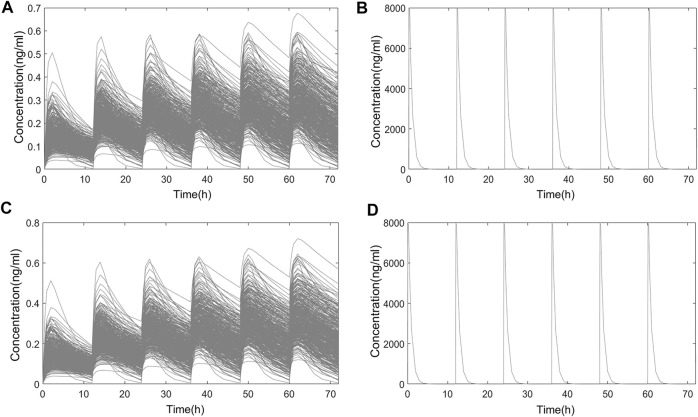
Simulated PK curve of multidose administration. **(A)** Systemic PK in Chinese; **(B)** nasal PK in Chinese; **(C)** systemic PK in Americans; **(D)** nasal PK in Americans.

In Chinese and Americans, the systemic PK and nasal tissue PK of OC-01 nasal spray 0.06 mg in single-dose administration and multidose administration (dosing interval 12 h) were separately simulated. The administration frequency and dosage followed the FDA Label (US Food and Drug Administration, 2005). The simulation of single-dose administration included PK data 72 h following single-dose administration. Regarding multidose administration, PK following administration for seven consecutive days was simulated. Only one dose was administered on day 7, and PK after 72 h was simulated. Simulated systemic PK and nasal PK following single-dose and multidose administration in the two populations are shown in Figure 6; Figure 7.

#### 3.4.1 Time to Steady-State

**FIGURE 8:**
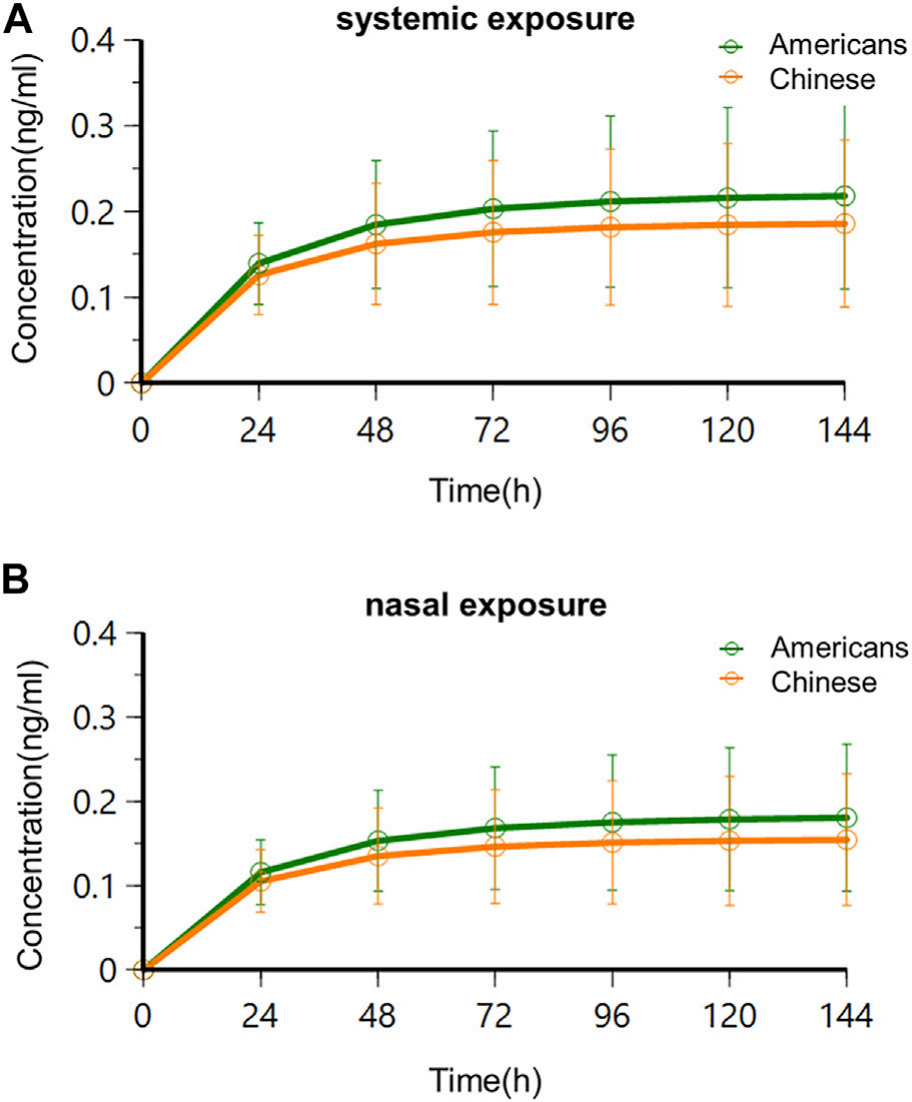
Trough concentration–time curves in Americans and Chinese following multidose administration. **(A)** Systemic exposure; **(B)** nasal exposure.

Based on the simulated results, systemic and nasal trough concentration–time curves in the two populations following multidose administration are shown in Figure 8.

The results showed that systemic and nasal drug concentrations reached the steady state following consecutive BID administration of 0.06 mg for 4 days in American and Chinese subjects. This agreed with the study results of oral varenicline formulation Chantix^®^. Clinical studies of Chantix^®^ in Western, Chinese, and Japanese subjects showed that PK exposure reached the steady-state 4 days following consecutive administration of varenicline at the recommended clinical oral dose (1 mg BID) (Xiao et al., 2009; Kikkawa et al., 2011; Nau et al., 2021). There was no significant difference of time to steady-state following BID administration of 0.06 mg between the two subject populations, and there was no significant difference of time to steady-state between systemic and nasal concentrations.

#### 3.4.2 Analysis of Steady-State Pharmacokinetics Following Single-Dose Administration and Multidose Administration

**FIGURE 9:**
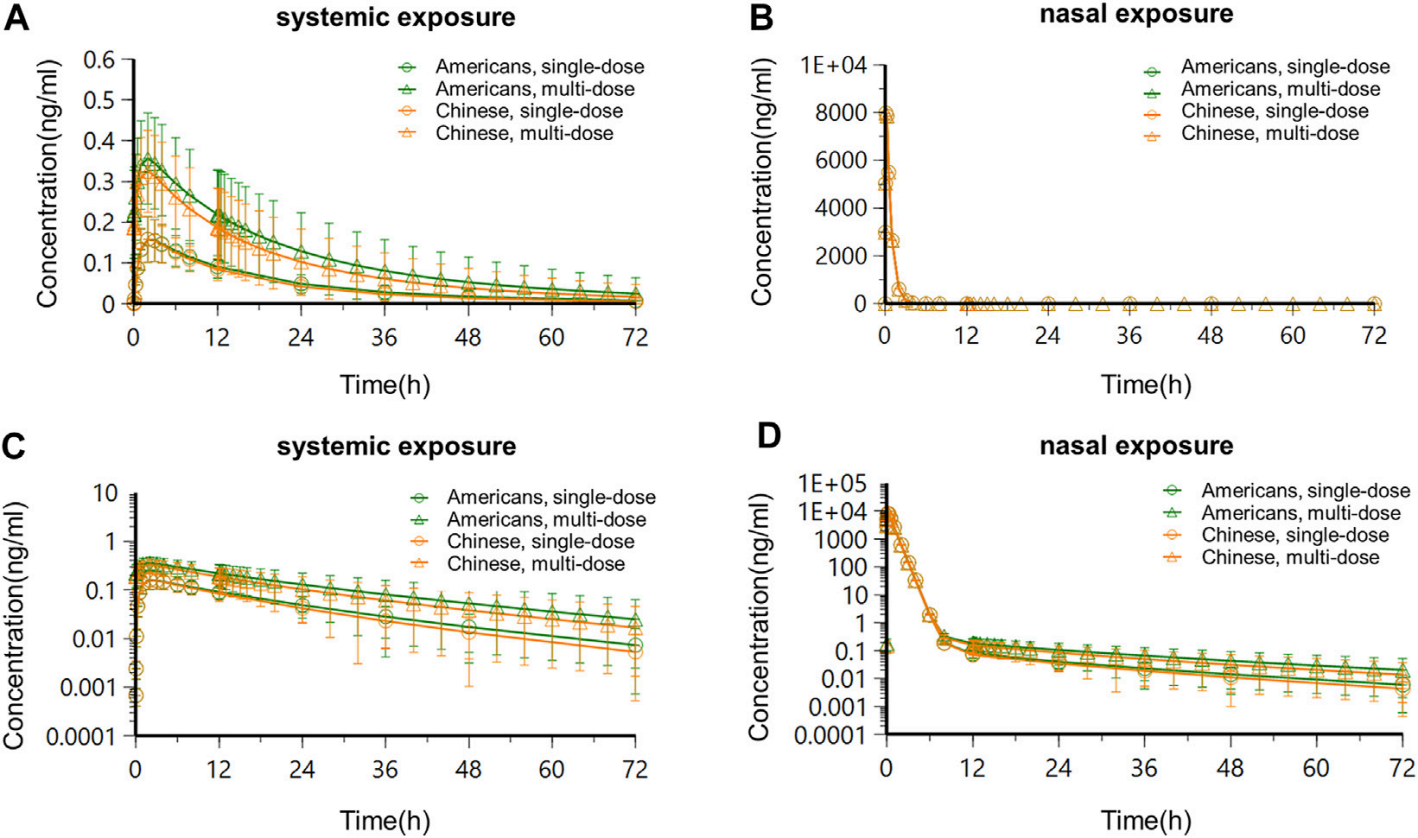
Concentration–time curves in Americans and Chinese after the steady-state following single-dose administration and multidose administration. **(A)** Systemic concentration–time curve-constant scale; **(B)** nasal concentration–time curve-constant scale; **(C)** systemic concentration–time curve-semi-logarithmic scale; **(D)** nasal concentration–time curve-semi-logarithmic scale.

Based on the semi-PBPK model, the simulated systemic and nasal PK exposures in the two populations in the steady-state following single-dose administration and multidose administration are shown in Figure 9. PK parameters are listed in Table 6.

**TABLE 6:** The simulated PK parameters in American and Chinese populations following single-dose administration and multidose administration of varenicline nasal spray, (a) systemic; (b) nasal.

Single-dose administration		Muilt-dose administration	
Ratio of Means (Chinese/Americans)	Americans Mean ± SD (CV%)	Chinese Mean ± SD (CV%)	Ratio of Means (Chinese/Americans)
1.00	2 (1–2)	2 (1–2)	1.00
1.00	0.36 ± 0.11 (31.31)	0.32 ± 0.1 (30.93)	0.89
0.97	3.47 ± 1.31 (37.86)	3.08 ± 1.18 (38.29)	0.89
0.88	9.41 ± 7.39 (78.59)	7.53 ± 5.89 (78.20)	0.80
NA	5.49 ± 7.56 (137.83)	3.99 ± 6.24 (156.35)	NA
0.87	15.39 ± 9.08 (58.99)	13.36 ± 7.88 (58.98)	0.87
0.98	162097.65 ± 47063.96 (29.03)	174458.65 ± 49971.71 (28.64)	1.08
1.13	10323.09 ± 7746.61 (75.04)	12661.31 ± 9146.65 (72.24)	1.23
NA	2.25	2.00	0.89
NA	2.38	2.17	0.91
1.00	0.08(0.08–0.08)	0.08 (0.08–0.08)	1.00
1.00	7983.94 ± 0.09 (0)	7983.91 ± 0.08 (0)	1.00
1.00	1817.02 ± 1.23 (0.07)	1816.65 ± 1.11 (0.06)	1.00
1.00	7164.89 ± 6.19 (0.09)	7163.29 ± 4.97 (0.07)	1.00
NA	0.01 ± 0.03 (239.85)	0.01 ± 0.02 (269.09)	1.00
0.87	15.38 ± 9.07 (58.97)	13.35 ± 7.87 (58.97)	0.87
0.87	185.68 ± 109.25 (58.84)	161.27 ± 94.92 (58.86)	0.87
1.00	8.37 ± 0.01 (0.09)	8.38 ± 0.01 (0.07)	1.00
NA	1.00	1.00	1.00
NA	1.00	1.00	1.00

	PK parameter	Unit	Americans Mean ± SD (CV%)	Chinese Mean ± SD (CV%)
Systemic Exposure	^a^ *T*_max_	h	2 (1–4)	2 (1–4)
	*C*_max_	ng/mL	0.16 ± 0.06 (35.23)	0.16 ± 0.06 (34.83)
	AUC_0-12_	h*ng/mL	1.46 ± 0.43 (29.46)	1.42 ± 0.41 (28.9)
	AUC_0-∞_	h*ng/mL	3.51 ± 1.37 (39.18)	3.1 ± 1.22 (39.19)
	AUC_%Extrap	%	5.58 ± 7.65 (137.2)	4.06 ± 6.32 (155.63)
	*T*_1/2_	h	15.4 ± 9.08 (58.97)	13.37 ± 7.88 (58.96)
	*V*/F	mL	378736.01 ± 154695.3 (40.85)	372023.3 ± 151874.14 (40.82)
	CL/F	mL/h	19856.41 ± 8191.77 (41.26)	22466.48 ± 9273.86 (41.28)
	R_*C*max_		NA	NA
	R_AUC0-12_		NA	NA
Nasal Exposure	^a^,^b^ *T*_max_	h	0.08 (0.08–0.08)	0.08 (0.08–0.08)
	*C*_max_	ng/mL	7983.76 ± 0 (0)	7983.76 ± 0 (0)
	AUC_0-12_	h*ng/mL	1815.12 ± 0.43 (0.02)	1815.08 ± 0.41 (0.02)
	AUC_0-∞_	h*ng/mL	7159.63 ± 1.15 (0.02)	7159.3 ± 1.03(0.01)
	AUC_%Extrap	%	0 ± 0.01 (173.02)	0 ± 0 (193.44)
	*T*_1/2_	h	15.37 ± 9.04 (58.83)	13.36 ± 7.84 (58.71)
	*V*/F	mL	185.77 ± 109.27(58.82)	161.47 ± 94.78 (58.7)
	CL/F	mL/h	8.38 ± 0 (0.02)	8.38 ± 0 (0.01)
	R*C*_max_		NA	NA
	R_AUC0-12_		NA	NA

NA, not applicable.

a *T*_max_ expressed as Median (Min-Max).

b Time to peak concentration (*T*_max_) after intranasal administration was the same in all simulated populations. The peak velocity after nasal administration was fast, and the simulated time points were the actual blood collection time points with short intervals. Therefore, no variability for *T*_max_ was observed. In addition, it was estimated that variability for *T*_max_ can be low, considering that the time to peak nasal concentration was short and the variability of parameters for nasal tissue in the PBPK model was low.

*C*_max_, maximum concentration; *T*_max_, time to *C*_max_.; AUC _0–12_, area under the concentration *vs*. time curve from the start of the last dose to12h; AUC_0-∞_, area under the plasma concentration–time curve from the start of the last dose to infinity; AUC_%Extrap, the proportion of the area under the plasma concentration–time curve from the last time at which there is a quantifiable concentration to infinity to AUC_0-∞_; *T*_1/2_, apparent terminal elimination half-life, *V*/F, apparent volume of distribution; CL/F, apparent total clearance; R_*C*max_, *C*_max_ at day 7/*C*_max_ at day1; R_AUC0-12_, AUC_0-12_ at day 7/AUC_0-12_ at day 1.

Following administration of OC-01 nasal spray of a single dose in Americans, the systemic concentration reached its peak at about 2 h, T_1/2_ was about 15.4 h, C_max_ was about 0.16 ng/ml, AUC_0-12_ was about 1.46 h*ng/mL, and CL/F and V/F were 19856.41 ml/h and 378736.01 ml, respectively. The nasal concentration reached its peak at about 0.08 h, T_1/2_ was about 15.37 h, C_max_ was about 7983.76 ng/ml, AUC_0-12_ was about 1815.12 h*ng/mL, and CL/F and V/F were 8.38 ml/h and 185.77 ml, respectively. When the same dose regimen was given to the Chinese subjects, it was indicated the systemic concentration reached its peak at about 2 h, T_1/2_ was about 13.37 h, C_max_ was about 0.16 ng/ml, AUC_0-12_ was about 1.42 h*ng/mL, and CL/F and V/F were 22466.48 ml/h and 372023.3 ml, respectively. The nasal concentration reached its peak at about 0.08 h, T_1/2_ was about 13.36 h, C_max_ was about 7983.76 ng/ml, AUC_0-12_ was about 1815.08 h*ng/mL, and CL/F and V/F were 8.38 ml/h and 161.47 ml, respectively (Table 6).

Following consecutive administration of 0.06 mg BID for 7 days in Americans, when the steady-state was reached on day 4, for each dose interval (*τ* = 12 h), the T_max_ was 2 h, T_1/2_ was about 15.39 h, C_max_ was about 0.36 ng/ml, AUC_0-12_ was about 3.47 h*ng/mL, and CL/F and V/F were 10323.09 ml/h and 162097.65 ml, respectively. After the steady-state was reached, apparent accumulation of exposure occurred, and the accumulation ratios R_Cmax_ and R_AUC0-12_ of systemic exposure were 2.25 and 2.38, respectively. The nasal concentration reached its peak at about 0.08 h, T_1/2_ was about 15.38 h, C_max_ was about 7983.94 ng/ml, AUC_0-12_ was about 1817.02 h*ng/mL, and CL/F and V/F were 8.37 ml/h and 185.68 ml, respectively. After the steady-state was reached, no apparent nasal exposure accumulated, and the accumulation ratios R_Cmax_ and R_AUC0-12_ were approximate to 1. At the same BID dose regimen, when the steady-state was reached in the Chinese population, the systemic concentration reached its peak at about 2 h, T_1/2_ was about 13.36 h, C_max_ was about 0.32 ng/ml, AUC_0-12_ was about 3.08 h*ng/mL, and CL/F and V/F were 12661.31 ml/h and 174458.65 ml, respectively. After the steady-state was reached, apparent accumulation of exposure occurred, and the accumulation ratios R_Cmax_ and R_AUC0-12_ of systemic exposure were 2.00 and 2.17, respectively. The nasal concentration reached its peak at about 0.08 h, T_1/2_ was about 13.35 h, C_max_ was about 7983.91 ng/ml, AUC_0-12_ was about 1816.65 h*ng/mL, and CL/F and V/F were 8.38 ml/h and 161.27 ml, respectively. After the steady-state was reached, no apparent nasal exposure accumulated, and the accumulation ratios R_Cmax_ and R_AUC0-12_ were approximately 1.

Overall, the systemic PK exposure of the drug showed apparent accumulation, with the systemic exposure accumulation ratios (R_Cmax_ and R_AUC0-12_) ranging between 2.00 and 2.38, in the steady-state following BID consecutive administration of 0.06 mg OC-01 nasal spray in American and Chinese subjects. Following single-dose and multidose administration in American and Chinese subjects, no significant difference was found in PK exposures (systemic and nasal). The ratios of the (Chinese subjects/American subjects) of the PK parameters ranged between 0.80 and 1.23.

#### 3.4.3 Ethnic Difference Evaluation

*In vitro* studies of varenicline (Faessel et al., 2006a; Faessel et al., 2010) showed that the drug was not metabolized by cytochrome P450 (CYP) enzymes which are mainly responsible for racial differences of drug metabolism. And the PK parameters (C_max_ and AUC_0-∞_) obtained from the clinical PK studies of oral varenicline in the Western, Chinese, and Japanese subjects were consistent (Supplementary Table S4) (Faessel et al., 2006a; Faessel et al., 2006b; Xiao et al., 2009; Kikkawa et al., 2011). Moreover, the pharmacokinetics of varenicline in OC-01 nasal spray was simulated using the semi-PBPK model. Based on the PK simulation of both single-dose and multiple-dose mentioned above, the intranasal and systemic PK characteristics in Chinese and Americans were also comparable. Therefore, it was convincing that the risk of ethnic difference of OC-01 nasal spray was low.

## 4 Discussion

For locally administered drugs that perform the therapeutic effects through systemic circulation, it is necessary to evaluate their systemic PK characteristics. But for these locally administered drugs which intend for local action, for example, nose, eye, and skin, determining local exposure of drug is significant, and whether to assess systemic exposure depends on actual situation. As stated in the guideline issued by NMPA (National Medical Products Administration, 2021), “It is recommended to focus on the assessment of local exposure after administration of locally applied and locally acting products”.

OC-01 (varenicline) nasal spray produces the effect at the target sites independent of systemic plasma concentrations. Thus, for OC-01 nasal spray, of which the active ingredient has been confirmed in previous studies of satisfactory safety (Xiao et al., 2009; Nau et al., 2021), the local nasal exposure is preferable to evaluate the PK property. However, it is difficult to acquire the drug exposure of local tissues, due to the limitation of technology in clinical PK study. As a result, the complex relationship between local nasal drug deposition and systemic drug plasma concentrations poses a significant challenge to investigators (Rygg et al., 2016), and hence, it is difficult to extrapolate the intranasal drug concentration from plasma concentration. Therefore, PBPK modeling, an essential tool in drug development, was utilized to predict the nasal exposure in human. However, few research studies of PBPK models are carried out domestically. In this study, a semi-PBPK model was established and validated using the data from varenicline after nasal and oral administration in Americans, as well as a single oral administration data in Chinese subjects. This well-validated model was used to simulate the systemic and the nasal exposure of OC-01 in Chinese following nasal spray administration.

It was indicated that intranasal concentrations of varenicline in subjects were significantly higher than systemic concentrations, suggesting the drug accumulated at the target site (nasal cavity), at the recommended dose (0.03 mg/spray/nostril, at a total dose of 0.06 mg per delivery, BID). The method of administration (nasal spray) accounted for varenicline accumulation at the nasal site. This finding matched with the studies in the literature (Frank et al., 2012; Cheng, 2014; Keeler et al., 2016), indicating that deposition of nasal sprays in the nasal cavity was common. Additionally, since the volume of nasal tissue was far smaller than the systemic distribution volume, the significantly higher drug concentration in the nasal cavity was reasonable. Besides, nasal PK exposure did not show apparent accumulation. This agreed with the study results of oral varenicline formulation Chantix^®^. Study results showed apparent PK accumulation in Western, Chinese, and Japanese populations given oral varenicline at the clinically recommended dose (1 mg BID). PK exposure showed apparent accumulation after the steady-state of plasma concentration was reached, with the accumulation fraction ranging between 1.93 and 2.70 (Xiao et al., 2009; Kikkawa et al., 2011).

The study results confirmed that PK profiles (systemic exposure and nasal exposure) of Chinese and Americans following single-dose administration and multidose administration of BID 0.06 mg OC-01 nasal spray were consistent with each other. After the steady-state was reached (4 days, BID), the concentration–time curves of systemic exposure and nasal exposure of two populations were consistent without significant difference after single-dose administration and multidose administration. Although PK exposure was slightly higher in Americans than in Chinese, the ratios of PK parameters ranged from 0.80 to 1.23, indicating no significant difference in PK exposure (systemic and nasal) between the two populations. Also, the systemic PK exposure showed apparent accumulation, with the accumulation ratio of systemic exposure ranging between 2.00 and 2.38. The results of clinical studies of Chantix^®^ single-dose and multiple-dose in the Western and Chinese populations suggest that varenicline has similar PK characteristics between the two populations, with no significant differences (Faessel et al., 2006a; Faessel et al., 2006b; Nau et al., 2021). According to the varenicline data in Americans (Nau et al., 2021), the systemic exposure of varenicline following intranasal administration (time to maximum concentration, 2 h) increased slightly faster than that following oral administration (time to maximum concentration, 4 h). This indicated that respiratory tract absorption played a vital role following intranasal administration. Since this nasal spray produced an effect at the trigeminal branch in the nasal cavity, its efficacy in Chinese and Americans could be better compared to local PK in nasal tissue. Moreover, the preclinical study of varenicline has shown that the drug is not a substrate of the CYP450 metabolic enzyme family (Faessel et al., 2006a; Faessel et al., 2010), implying a negligible risk of racial differences between Chinese and Americans.

The present study successfully indicated that the semi-PBPK model, established based on the anatomy and physiology of human respiratory tract (Figure 1), could give reasonable simulations of the distribution and absorption of the nasal spray drug at different segments. Therefore, a mechanism-based respiratory model provides an alternative for explaining and simulating drug absorption after intranasal administration, and it provides viable framework for other novel nasal spray drugs. Notably, this semi-PBPK model is still an evolving model and should be updated with additional physiological and specific PK data. Moreover, application of this model for additional drugs may further demonstrate its strengths and weaknesses.

## 5 Conclusion

In conclusion, we established and validated a nasal semi-PBPK model successfully to predict the system and nasal pharmacokinetics of OC-01(varenicline) in Chinese population. At the recommended clinical dose (0.03 mg/spray/nostril, at a total dose of 0.06 mg per delivery, BID), the systemic and the nasal exposure following the administration of OC-01 nasal spray had no significant difference between the Chinese and Americans. And the risk of ethnic differences between Americans and Chinese was low. Therefore, it was recommended not to carry out phase I clinical PK study in Chinese subjects with DED and to conduct phase III clinical study directly. This nasal inhalation semi-PBPK model can also be used to develop novel nasal spray drugs in the future.

## Data Availability

The original contributions presented in the study are included in the article/Supplementary Material, further inquiries can be directed to the corresponding author.
