# Supplementary material for: Semi-PBPK Modeling and Simulation to Evaluate the Local and Systemic Pharmacokinetics of OC-01(Varenicline) Nasal Spray
Source: Front Pharmacol. 2022 Jul 7;13:910629. doi: 10.3389/fphar.2022.910629 (PMC9301199; doi:10.3389/fphar.2022.910629)
Supplement: Supplementary file 1 [file DataSheet1.docx]

Supplementary Material

**Table S1** The equations used to describe the mass change of drug in each segment of mucus (assuming instantaneous dissolution).

**Table S2** The equations used to describe the mass change of drug in tissue.

**Table S3** The equations used to describe the mass change of drug in pharynx and systemic circulation.

**Table S4** The PK parameters in western, Chinese and Japanese following single-dose and multi-dose administration of varenicline.

**Table S1** The equations used to describe the mass change of drug in each segment of mucus (assuming instantaneous dissolution).

| **No.** | **Equations** |
| --- | --- |
| 1 | $\frac{\text{d}\text{M}_{\text{nose-diss}}}{\text{dt}}\text{ = - }\text{PS}_{\text{nose}}\text{*(}\frac{\text{M}_{\text{nose-diss}}}{\text{V}_{\text{nose-ELF}}}\text{*}\text{f}_{\text{u,ELF}}\text{ - }\frac{\text{M}_{\text{nose-diss}}}{\text{V}_{\text{nose-tissue}}}\text{*}\text{f}_{\text{u,tissue}}\text{)}$ |
| 2 | $\frac{\text{d}\text{M}_{\text{BB-diss}}}{\text{dt}}\text{ = -}\text{k}_{\text{MCC}}\text{*}\text{M}_{\text{BB-diss}}\text{ +}\text{k}_{\text{MCC}}\text{*}\text{M}_{\text{bb-diss}}\text{- }\text{PS}_{\text{BB}}\text{*(}\frac{\text{M}_{\text{BB-diss}}}{\text{V}_{\text{BB-ELF}}}\text{*}\text{f}_{\text{u,ELF}}\text{ - }\frac{\text{M}_{\text{BB-diss}}}{\text{V}_{\text{BB-tissue}}}\text{*}\text{f}_{\text{u,tissue}}\text{)}$ |
| 3 | $\frac{\text{d}\text{M}_{\text{bb-diss}}}{\text{dt}}\text{ = -}\text{k}_{\text{MCC}}\text{*}\text{M}_{\text{bb-diss}}\text{- }\text{PS}_{\text{bb}}\text{*(}\frac{\text{M}_{\text{bb-diss}}}{\text{V}_{\text{bb-ELF}}}\text{*}\text{f}_{\text{u,ELF}}\text{ - }\frac{\text{M}_{\text{bb-diss}}}{\text{V}_{\text{bb-tissue}}}\text{*}\text{f}_{\text{u,tissue}}\text{)}$ |
| 4 | $\frac{\text{d}\text{M}_{\text{AL-diss}}}{\text{dt}}\text{ = - }\text{PS}_{\text{AL}}\text{*(}\frac{\text{M}_{\text{AL-diss}}}{\text{V}_{\text{AL-ELF}}}\text{*}\text{f}_{\text{u,ELF}}\text{ - }\frac{\text{M}_{\text{AL-diss}}}{\text{V}_{\text{AL-tissue}}}\text{*}\text{f}_{\text{u,tissue}}\text{)}$ |

M_nose-diss_, the mass of the drug dissolved in nose mucus; M_BB-diss_, the mass of the drug dissolved in bronchi mucus; M_bb-diss_, the mass of the drug dissolved in bronchioles mucus; M_AL-diss_, the mass of the drug dissolved in alveoli mucus; V, Volume; f_u, ELF_, free fraction of drug in ELF; f_u, tissue_, free fraction of drug in tissue; PS, permeation elimination rate.

**Table S2** The equations used to describe the mass change of drug in tissue.

| **No.** | **Equations** |
| --- | --- |
| 1 | $\text{V}_{\text{nose-tissue}}\frac{\text{d}\text{C}_{\text{nose-tissue}}}{\text{dt}}\text{ = }\text{PS}_{\text{nose}}\text{*(}\text{C}_{\text{nose-ELF}}\text{*}\text{f}_{\text{u,ELF}}\text{ - }\text{C}_{\text{nose-tissue}}\text{*}\text{f}_{\text{u,tissue}}\text{) - }\text{PS}_{\text{nose}}\text{*(}\text{C}_{\text{nose-tissue}}\text{*}\text{f}_{\text{u,tissue}}\text{ - }\text{C}_{\text{p}}\text{*}\text{f}_{\text{u,p}}\text{)}$ |
| 2 | $\text{V}_{\text{BB-tissue}}\frac{\text{d}\text{C}_{\text{BB-tissue}}}{\text{dt}}\text{ = }\text{PS}_{\text{BB}}\text{*(}\text{C}_{\text{BB-ELF}}\text{*}\text{f}_{\text{u,ELF}}\text{ - }\text{C}_{\text{BB-tissue}}\text{*}\text{f}_{\text{u,tissue}}\text{) - }\text{PS}_{\text{BB}}\text{*(}\text{C}_{\text{BB-tissue}}\text{*}\text{f}_{\text{u,tissue}}\text{ - }\text{C}_{\text{p}}\text{*}\text{f}_{\text{u,p}}\text{)}$ |
| 3 | $\text{V}_{\text{bb-tissue}}\frac{\text{d}\text{C}_{\text{bb-tissue}}}{\text{dt}}\text{ = }\text{PS}_{\text{bb}}\text{*(}\text{C}_{\text{bb-ELF}}\text{*}\text{f}_{\text{u,ELF}}\text{ - }\text{C}_{\text{bb-tissue}}\text{*}\text{f}_{\text{u,tissue}}\text{)- }\text{PS}_{\text{bb}}\text{*(}\text{C}_{\text{bb-tissue}}\text{*}\text{f}_{\text{u,tissue}}\text{ - }\text{C}_{\text{p}}\text{*}\text{f}_{\text{u,p}}\text{)}$ |
| 4 | $\text{V}_{\text{AL-tissue}}\frac{\text{d}\text{C}_{\text{AL-tissue}}}{\text{dt}}\text{ = }\text{PS}_{\text{AL}}\text{*(}\text{C}_{\text{AL-ELF}}\text{*}\text{f}_{\text{u,ELF}}\text{ - }\text{C}_{\text{AL-tissue}}\text{*}\text{f}_{\text{u,tissue}}\text{)- }\text{PS}_{\text{AL}}\text{*(}\text{C}_{\text{AL-tissue}}\text{*}\text{f}_{\text{u,tissue}}\text{ - }\text{C}_{\text{p}}\text{*}\text{f}_{\text{u,p}}\text{)}$ |

C, concentration of drug in tissue; f_u,p_, free fraction of drug in plasma

**Table S3** The equations used to describe the mass change of drug in pharynx and systemic circulation.

| **No.** | **Equations** |
| --- | --- |
| 1 | $\frac{\text{d}\text{M}_{\text{ET}}}{\text{dt}}\text{ = - }\text{k}_{\text{a}}\text{*}\text{M}_{\text{ET}}\text{ + }\text{k}_{\text{MCC}}\text{*}\text{M}_{\text{BB-diss}}$ |
| 2 | $\text{V}_{\text{p}}\frac{\text{d}\text{C}_{\text{p}}}{\text{dt}}\text{ = }\text{k}_{\text{a}}\text{*}\text{M}_{\mathrm{ET}}\text{ + }\text{PS}_{\text{nose}}\text{*(}\text{C}_{\text{nose-tissue}}\text{*}\text{f}_{\text{u,tissue}}\text{ }\text{-}\text{ }\text{C}_{\text{p}}\text{*}\text{f}_{\text{u,p}}\text{)+}\text{PS}_{\text{BB}}\text{*(}\text{C}_{\text{BB-tissue}}\text{*}\text{f}_{\text{u,tissue}}\text{ - }\text{C}_{\text{p}}\text{*}\text{f}_{\text{u,p}}\text{)}\text{}$ ${\text{+}\text{PS}}_{\text{bb}}\text{*(}\text{C}_{\text{bb-tissue}}\text{*}\text{f}_{\text{u,tissue}}\text{ - }\text{C}_{\text{p}}\text{*}\text{f}_{\text{u,p}}\text{)+ }\text{PS}_{\text{AL}}\text{*(}\text{C}_{\text{AL-tissue}}\text{*}\text{f}_{\text{u,tissue}}\text{ - }\text{C}_{\text{p}}\text{*}\text{f}_{\text{u,p}})\text{-}\text{ }\text{CL}\text{*}\text{ }\text{C}_{\text{p}}$ |

M, mass of drug in tissue.

**Table S4** The PK parameters in western, Chinese and Japanese following single-dose and multi-dose administration of varenicline.

| Dose group | PK parameters（Unit） | Western | Chinese | Japanese |
| --- | --- | --- | --- | --- |
| Single-dose administration (1 mg) | C_max_ (ng/mL) | 6.20 ± 1.10 | 4.95 ± 0.69 | 4.97 ± 0.56 |
|  | AUC_0-∞_ (h^*^ng/mL) | 102 ± 14 | 91.36 ± 21.23 | 104 ± 11 |
|  | T_1/2_ (h) | 13.6 ± 6.1 | 15.23 ± 3.38 | 18.4 ± 3.2 |
|  | ^#^T_max_ (h) | 3.00 ± 2.16 | 3.00 (0.50-6.00) | 3.0 (2.0-4.0) |
|  | CL_r_ (mL/min) | NA | 115.0 ± 66.7 | 119 ± 35 |
| Steady state following multi-dose administration (1 mg BID) | C_max_ (ng/mL) | 10.2 ± 1 | 9.57 ± 1.54 | 12.0 ± 2.9 |
|  | AUC_0-12_(h^*^ng/mL) | 105 ± 16 | 87.48 ± 17.07 | 116 ± 29 |
|  | T_1/2_ (h) | 31.5 ± 7.7 | 18.34 ± 3.61 | 24.2 ± 3.5 |
|  | ^#^T_max_ (h) | 2.0 (1.0-4.0) | 2.5 (1-6) | 3.0 (2.0-4.0) |
|  | CL_r_ (mL/min) | 125 ± 54 | 100.8 ± 55.7 | 90.5 ± 20.0 |
|  | R_Cmax_ | 2.50 | 1.93 | 2.27 |
|  | R_AUC0-12_ | 2.67 | 2.10 | 2.72 |

^#^T_max_ expressed as Mean±SD in western and Median (Min-Max) in Chinese and Japanese.

NA, missing value.
